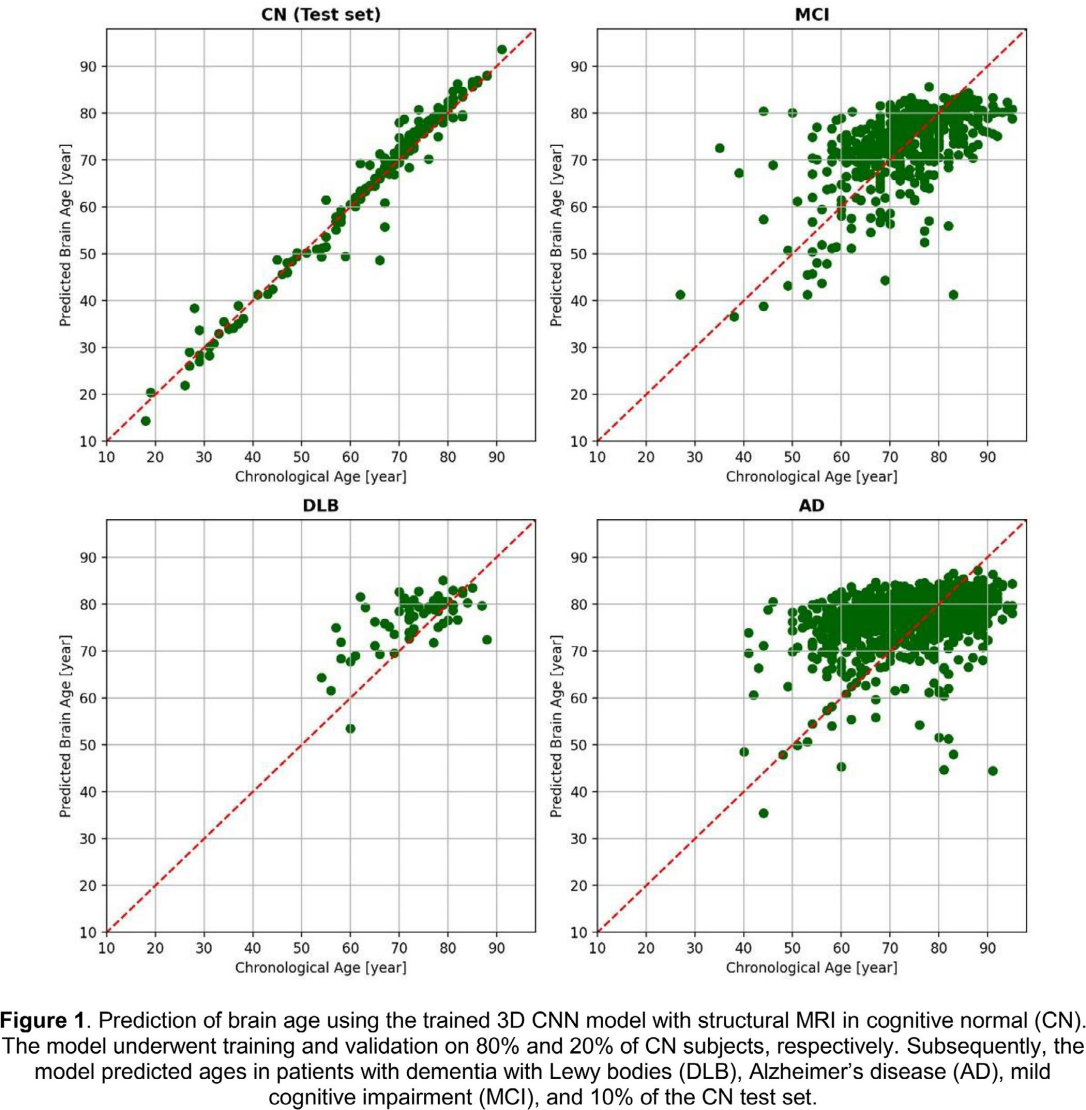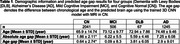# Advanced brain age prediction using 3D convolutional neural network on structural MRI

**DOI:** 10.1002/alz.089776

**Published:** 2025-01-09

**Authors:** Babak Ahmadi, Zohreh Morshedizad, Mostafa Reisi Gahrooei, Abbas Babajani‐Feremi

**Affiliations:** ^1^ University of Florida, Gainesville, FL USA

## Abstract

**Background:**

Predicting brain age from neuroimaging data is an emerging field. The age gap (AG), the difference between chronological age (CA) and brain age (BA), is crucial for indicating individual neuroanatomical aging. Previous deep learning models faced challenges in generalizability and neuroanatomical interpretability. We incorporated patients with different dementia types, including dementia with Lewy bodies (DLB) and Alzheimer’s disease (AD), alongside mild cognitive impairment (MCI) and cognitive normal (CN) individuals. This inclusive strategy is essential for comprehensive mapping of neurocognitive trajectories and understanding distinct aging patterns across various cognitive conditions.

**Method:**

Utilizing T1‐weighted MRI images of n = 3,859 subjects (Table 1) from the CamCAN, NACC, and ADNI databases, this study aimed to predict brain age in four groups (CN, MCI, AD, and DLB). Structural MRI data were spatial normalized and skull‐striped. Then a 3D Convolutional Neural Network (CNN) based on the skull‐striped data was used for age prediction. The model’s architecture includes three convolutional layers with ReLU activation, max‐pooling, batch normalization, and dropout for regularization, ending with global average pooling and dense layers. The model was trained and validated on CN subjects. The trained model was used to predict age in MCI, DLB, and AD patients as well as the test set of CN subjects.

**Result:**

The 3D CNN model accurately predicted brain age in the CN test set with an AG of 0.64 ± 2.74 years and an absolute AG of 1.86 ± 2.11 years (Figure 1 and Table 1). In DLB and AD patients, the average AG was 3.81 and 2.90 years, respectively, and significantly larger than 0 (P < 10^‐5^), indicating accelerated aging patterns in these groups. The average AG of MCI was 0.09 years which was significantly smaller than that of both DLB and AD (P < 10^‐3^), indicating the early stage of impairment in MCI patients.

**Conclusion:**

Our 3D CNN model accurately predicted brain age in cognitively normal individuals and identified accelerated aging in DLB and AD patients. The model's precision highlights its potential for early detection and understanding of neurocognitive trajectories, contributing to advancements in neurological research and clinical diagnostics.